# Humeral Tip-apex-distance as a Prognostic Marker for Proximal Humeral Fractures in 203 Patients

**DOI:** 10.2174/1874325001711010297

**Published:** 2017-04-20

**Authors:** Dominik Saul, Tobias Himmelmann, Klaus Dresing

**Affiliations:** 1 Department of Trauma, Orthopaedics and Reconstructive Surgery, Georg-August-University of Goettingen, Goettingen, Germany; 2 MVZ Prof. Uhlenbrock u. Partner, Dortmund, Germany

**Keywords:** Humeral head fracture, Humeral tip-apex-distance, HTAD, Philos^®^ plate, Calcar, Loss of fixation, Postoperative treatment

## Abstract

**Background:**

Humeral head fractures and their postoperative outcome remain a challenging problem in surgical daily routine. Predictive factors for loss of fixation are rare.

**Objective:**

Determination of predictive factors for the failure of osteosynthesis with the loss of fixation or migration of screws in humeral head fractures.

**Method:**

From 1995 to 2011, 408 patients with proximal humeral fractures [mean age 66.6 years, 50.9-82.3 years] and osteosynthesis were analyzed. Two hundred and three received open reduction internal fixation (ORIF) with the PHILOS^®^ plate. The non-locking plate was used in 80, the locking plate in 16 and humeral head prosthesis in 26 patients, in addition to 23 patients undergoing other procedures. Intraoperative reduction that achieved an anatomical alignment of the medial aspect of the humerus (humeral calcar) was assessed in 94 patients by postoperative X-ray analysis. The loss of fixation was evaluated by a follow-up of three to five X-rays and measurement of the humeral tip-apex-distance (HTAD).

**Results:**

For stable fixed fractures with an intact calcar, percentual HTAD was significantly higher than for unstable fixed fractures (p=0.04). Morbidity, such as hypertension, orthopedic operations or diabetes, strongly influenced the HTAD, while postoperative passive motion treatment modestly affected the HTAD over time.

**Conclusion:**

The anatomic reconstruction of the calcar, leading to stable fixation of humeral head fractures, can significantly prevent an overproportioned decrease in the HTAD in postoperative X-rays and seems to be vital in multimorbid patients. Measurement of the HTAD over time delivers a tool for early detection of secondary loss of fixation.

## INTRODUCTION

Fractures of the proximal humerus account for approximately 6% of all adult fractures [1]. Some (distinct) undisplaced fractures may be sufficiently treated with conservative therapy [2], while displaced and unstable fractures need to be treated surgically [3, 4]. Remarkably, there is an evidence that dislocated three- and four-party fractures may be sufficiently treated conservatively [5]. A meta-analysis, nonetheless, questioned the benefits of surgery after one or two years [6]. According to Zhu *et al.*, locking plate fixation leads to a better functional outcome compared to the locking nail approach [7]. The loss of surgical fixation depends on the age, local bone morphogenic density (BMD) and anatomic reduction, especially of the medial cortical support/calcar [8-12]. After surgery, predictable factors of implant failure with loss of fixation are rare, and radiographic detection of cut-out [13] and osteonecrosis, especially in complex fractures [14-16], remains challenging.

In trochanteric fractures of the hip, measuring the tip-apex distance is a valid tool for describing the position of the screw [17] and to predict the potential cut-out [18-22]. Therefore, we developed an easy approach to assess the plate position after open reduction and internal fixation (ORIF) in proximal humeral fractures, which is comparable to the tip-apex distance in proximal femoral fractures.

The objective of this retrospective study was to determine whether the humeral tip-apex-distance (HTAD) in humeral plate osteosynthesis was a reliable factor for assessing the outcome of these fractures and a predictor of postoperative failure, such as cut-out.

## MATERIALS AND METHODS

### Patient Cohort and Surgery Approaches

Patients with proximal humerus fractures admitted to our hospital (university trauma center level-1 in Germany) from 1995 to 2011, were retrospectively investigated. Inclusion criteria were:

- Radiologically verified proximal humeral fractures
- Complete recordings
- Complete X-ray follow-up
- Age >18 years

An additional inclusion criterion for the HTAD measurement was:

- surgical treatment with the PHILOS^®^-plate.

Four hundred and eight patients fulfilled the criteria. Filemaker-datasets were built based upon operation protocols.

### Assessed Parameters

Patient characteristics:

1. Age
2. Gender
3. BMI (kg/m^2^)
4. Pre-existing morbidities
5. Causes of accident

Classification:

1. Soft tissue injury according to Tscherne/Oestern [23] and Gustilo/Anderson [24, 25].
2. The type of fracture was classified according to Neer [26] and Müller/AO [27].

Reduction/treatment:

1. Open or closed anatomical reduction
2. Technique: PHILOS^®^ plate (Synthes, Switzerland), humeral head prosthesis, intramedullary nailing, and non-locking plate
3. Humeral reduction in all planes
4. Reduction of the collum chirurgicum, the so-called “calcar of the humerus”
5. Time of operation
6. Valgus/varus malalignment

Perioperative treatment:

1. Reduction and immobilization
2. Extended ambulant physiotherapy and
3. Use of a continuous-passive-motion-splint (CPM)

### Humeral Tip-Apex-Distance (HTAD)

**Fig. (1):**
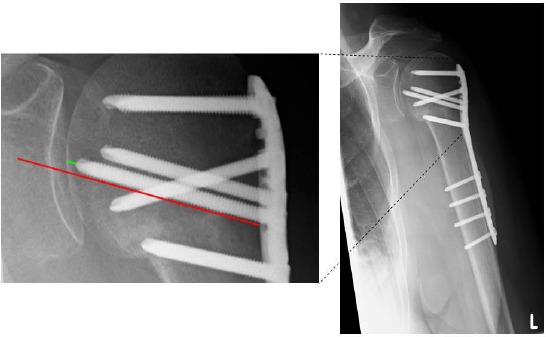
Measurement of the humeral tip-apex-distance (HTAD). Parallel to the screw-axis (red line) and beginning on the tip to the medial edge of the humeral corticalis (green line), the distance was scaled to the outer cortical limit.

X-ray imaging was conducted during five different time points (on average, 13.01, 64.78, 178.75, 213.34, and 165.80 days after accident) and in at least two X-ray planes. The angle could be confirmed by the great and minor tubercle and location of the osteosynthesis material. Right angle screws could be scaled the whole length (Fig. **1**).

The humeral tip-apex-distance was defined based on Baumgaertner *et al.* for femoral head fractures [19]. The authors defined the TAD as the distance in mm from the tip of the screw to the outer cortical limit of the femoral head in extension of the screw. Alteration in the TAD was considered proof of a positional change in the bone. We applied this definition for the tip-apex-distance of most cranial screws in the humeral head. Parallel to the screw-axis, a second line was drawn for scaling the distance from the medial edge of humeral corticalis to the tip of the screw (Fig. **1**).

### Patient Stratification

Operative reduction was evaluated in 94 patients (age and fracture type were equal to the overall collective due to one-way ANOVA). The criterion for this special sub-collective was a representative postoperative X-ray in which anatomical reduction could be sufficiently assessed. Two criteria were considered:

1. Correct axial and anatomical alignment
2. Reduction and restoration of the medial cortex (medial collum chirurgicum, “calcar” humeri)

Definition of sufficient reduction was documented with criteria 1+2; otherwise, the reduction was classified as “insufficient”.

Patients were re-evaluated after two weeks, two months, six months and eight months with X-ray examination. However, each patient’s compliance resulted in variable time points for the X-rays.

Postoperative treatment was generally performed with limited abduction and passive motion for six weeks, which was followed by functional training by physiotherapists.

### Data Analysis

Statistical analysis and graphical assessment were performed with GraphPad Prism (version 5.04, GraphPad Software, La Jolla California USA).

The following parameters were used: standard deviation, mean, median and ANOVA. P-values ≤0.05 were set as statistically significant.

## RESULTS

Four hundred and eight patients were included in this study. Their mean age was 66.62 ±15.72 [50.9-82.3] years; 63.54% were female, and 36.46% were male.

**Fig. (2):**
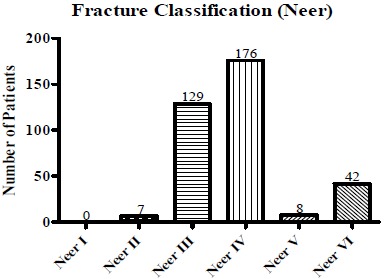
**Neer classification of the patient cohort.** Fracture classification according to Neer shows predominantly types IV and III in the investigated cohort, accounting for 85% fractures.

**Fig. (3):**
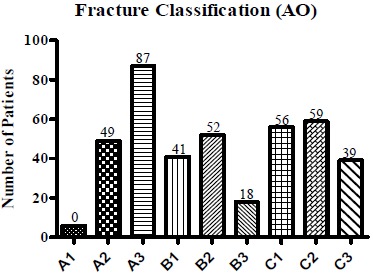
**Müller/AO classification of the patient cohort.** Fracture classification according to Müller/AO primarily shows A3, C2 and C1 fractures.

Fracture classification according to Neer and Müller/AO is shown in Fig. (**2**) and Fig. (**3**). In most cases, Neer III and IV (Fig. **2**) and AO A3 and C2 (Fig. **3**) fractures could be detected.

## Cause of Accident

**Fig. (4):**
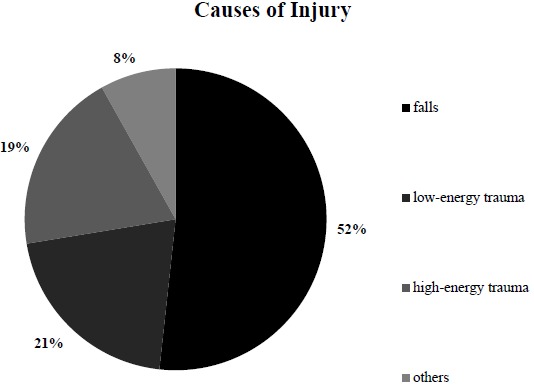
**Causes of injury in the investigated group.** Causes of injury were commonly falls (52%), which was followed by low-energy trauma in 21%.

When analyzing the causes of injury, we found that most accidents could be attributed to a fall, which was the case in 210 patients (51.72%); low-energy trauma was observed in 84 (20.69%) patients, and high-energy trauma was observed in 79 (19.46%) patients, which was followed by 33 (8.13%) patients who had another cause of injury (Fig. **4**).

## Comorbidities

Comorbidities were evaluated and were highly heterogeneous. We detected hypertension in 94 patients (23.04%), obesity in 44 (10.78%), an apoplectic insult in 25 patients (6.13%), malignancies in 27 patients (6.62%) and alcohol abuse in 31 patients (7.6%), while 65 had other comorbidities (15.94%).

## Postoperative Treatment

**Fig. (5):**
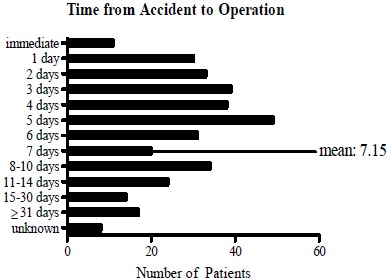
**Measured time from fracture to operative treatment.** The time from accident to operation was found to be on average of 7.15 days; the mode was five days.

After admission to the hospital, the elapsed time from accident to operation was an average of 7.15 days and could be divided into several time frames. For most patients, up to 5 days were needed to perform the surgery following injury. By contrast, few individuals were treated later than 15 days after their accident (Fig. ** 5**).

## Operative Procedure

**Fig. (6):**
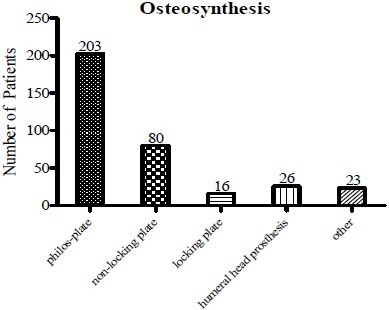
**Different types of ORIF.** Osteosynthesis was performed by PHILOS^®^-Plate in most cases, which was followed by a non-locking plate.

Finally, surgical techniques have been compared among the patients. Osteosynthesis was primarily performed by PHILOS^®^-Plate in 203 cases (58.33%), non-locking plate in 80 patients (22.99%), locking plate in 16 patients (4.6%) and humeral head prosthesis in 26 patients (7.47%); 23 underwent other operational procedures (6.61%) (Fig. **6**).

The representative subgroup investigated for reduction consisted of 94 patients; 49 were considered sufficiently treated (52.13%). On the other hand, 45 patients (47.87%) were considered to be insufficiently supplied.

## Humeral Tip-Apex-Distance

**Fig. (7):**
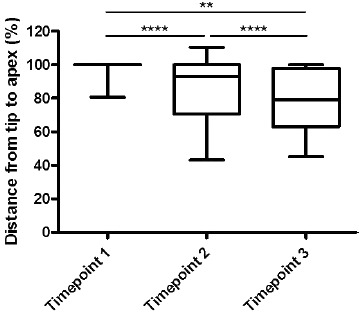
**Humeral tip-apex-distance (HTAD) determined at three time points.** From the beginning (Timepoint 1) to the third measurement (Timepoint 3), the distance from the tip to apex increased, revealing significant differences between timepoints 1 and 3.

In all X-rays, the mean tip-apex distance was measured. As expected, the first X-ray (taken an average of 13.1 days after the accident) revealed an HTAD of 99.93%. During the second X-ray, no significant difference was found (101.66%; an average of 64.78 days after the accident), while values of 85.86% (178.75) could be detected in the third, 83.04% (213.34) in the fourth and 60.21% (165.80) in the fifth measurements (Fig. **7**).

## Humeral Tip-Apex -Distance in Stable/Instable Reduction

**Fig. (8):**
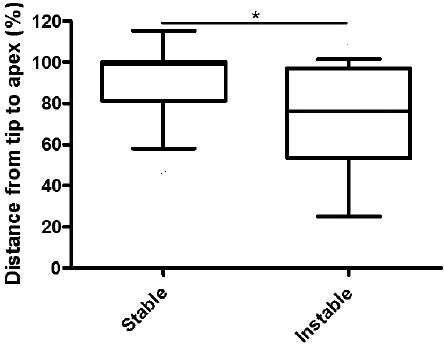
**Humeral tip-apex-distance (HTAD) over time in stable vs. instable reduced fractures.** The relationship from the first to the third X-ray is significantly higher (lower difference in the measured HTAD) in a stable situation compared to an instable situation.

The measured tip apex distance was significantly greater from the first to the third X-ray in patients with radiologically stable osteosynthesis (95.41%±4.753) compared to those with instable fixation (77.69%±7.093, p=0.0443), (Fig. **8**).

## Humeral Tip-Apex-Distance and Comorbidities

Morbidities were analyzed, including the number of comorbidities and their combinations (Table **1**).

An adequate reduction, short time of surgery and providing continuous passive motion treatment (CPM) significantly affected the HTAD (Table **1**).

## DISCUSSION

Proximal humeral fractures in the elderly have a poor outcome, and open reduction with locking plate osteosynthesis accounts for substantial complication rates that range from 49% [12]up to 76% [28-32], with secondary dislocation/cutting out as one major problem [33]. Although intraoperative failures are frequently preventable [34], postoperative X-rays are often difficult to evaluate, and comparison remains challenging [13, 35, 36], especially because missing cortical support causes a poor outcome [37]. Although some key aspects of postoperative failure have been identified, a simple approach to evaluating the postoperative result remains to be elucidated.

By measuring the humeral tip-apex-distance, a simple approach for quantifying the postoperative status is presented. A shortage in the HTAD means there is convergence of the screw-tip to the outer bone limit, and an increase indicates removal of the screw-tip from the outer bone limit.

Because the HTAD of the first X-ray is normally defined as 100%, the distance determined during the following X-rays normally falls below 100%, but it may also exceed 100% because of imaging variations. From the first to the third measurement, the best results could be found in this study.

Our results demonstrate a physiological sintering of head fragments in all patients provided with osteosynthesis of the first screw over time, which was indicated by an increasing HTAD - and decreasing distance percentage in relation to the first X-ray distance - from the first to the third X-ray.

In our study, two-part fracture was the most prevalent type, as was similarly demonstrated by Euler *et al.* [16] Consequently, the most common operative procedure was plate osteosynthesis (85.9%), which was followed by humeral head prosthesis (7.5%); these results are comparable to the retrospective study by Katthagen *et al.* [38].

While the average age in our cohort was 66.62 years, which is very close to a Swedish study (66.8), and falls were the main cause of injury in both studies, the gender was predominantly female (1.74:1), which is consistent with a large study in which women had a higher risk of fractures in the humerus than men [39] as well as with a great prospective multicenter study [40].

If surgery was performed more than 5 days after the accident, an increased risk of complications could be observed [41]. In our population, 57.5% of interventions were conducted during this critical time period. Data on the ideal time of surgery are rare. Südkamp *et al.* reported an average time of 4.1±3.4 days [40] from injury to operation, but the authors did not correlate the time with the outcome.

Because “cutting out” remains a major issue the surgeon should consider, and up to 14% of patients suffer from screw perforation, especially in ankle-stable plates [40], regular postoperative radiologic controls are obligatory; however, evaluation remains difficult. Several parameters, such as varus displacement or medial comminution, were significantly correlated with reduction loss [10, 42], but more specific parameters are needed to detect postoperative failures early.

In our population, patients with an instable fixation, especially those missing cortical support at the calcar, tend to a greater HTAD in postoperative X-rays. The cortical support on the medial column is of great importance for bone healing in the proximal humerus [43], especially in complex fractures [31, 42, 44-46]. The HTAD seems to illustrate the importance of a stable reduction in operative treatment because it is proportionally greater in the early postoperative radiographs if the medial column is not correctly fixed. Further parameters correlated with a greater HTAD, such as morbidity, could act as surrogate parameters for low bone quality or low patient compliance. We observed that multiple comorbidities significantly affected the HTAD. While age lower or higher than 65 years did not affect the HTAD, neither did the type of accident, polytrauma, BMI, gender, time between accident and admission to hospital/operation, osteoporosis, beginning of mobilization, locking plate vs. non-locking plate, physiotherapy or period of hospitalization.

Because it remains to be determined which group of patients benefits from implant augmentation [16, 47, 48], especially in angle stable osteosynthesis [33], some suggest multifragmentary fractures and elderly patients with a low BMD should be considered for this procedure [8]. From our point of view, we recommend patients with multiple comorbidities or fragmented calcar, as a result of the greater HTAD over time in this special subgroup, should be considered for the procedure. Such treatment could prevent the growing HTAD, which should be further analyzed in future studies.

One major limitation of this study is the variation in the measurement time. The HTAD highly depended on patients presenting for follow up, which was influenced by varying compliance in the studied group.

Moreover, we could demonstrate that a short time before operation and providing CPM help to maintain a small HTAD. Although data on the former is rare, we presume that a shorter time before surgery correlates with simple fractures and is therefore more likely to end in a stable fixation, which is in agreement with Yang *et al.* [45]. The latter is consistent with data in the literature [49-51].

## CONCLUSION

Taken together, this study illustrates the importance of anatomical reduction, especially on the medial corticalis, and the benefit of measuring the HTAD in postoperative X-rays. This measurement can help evaluate the status of fixation and detect early loss of reduction, allowing for prompt decisions about when to intervene and when to practice watchful waiting.

Based on this study, we recommend regular X-ray controls with subsequent recording of the HTAD and passive mobilization by CPM.

## Figures and Tables

**Table 1:** Parameters investigated in relation to the HTAD and their predictive value.

Parameters investigated	p-value (HTAD)
HTAD	0.0001 ****
Morbidity and Osteoporosis	
Morbidity (pre-existing illness or not)	0.0012 **
Morbidity (1 vs. 2 vs. 3 vs. 4 vs. 5)	0.8553
Morbidity (hypertension, orthopedic operations, and diabetes)	0.0106 *
Morbidity (coronary heart disease, hypertension, post myocardial infarction, and diabetes)	0.0409 *
Morbidity (tumor disease, hypertension, and diabetes)	0.0220 *
Osteoporosis	0.9576
Circumstances and patient characteristics	
Type of accident	0.0900
Polytrauma	0.4037
BMI	0.5142
Gender	0.0695
Age (<65 vs. >65 years)	0.1803
Time until operation, stay in the hospital	
Time until admission	0.6105
Time between accident and operation	0.5014
Stay in the hospital (<1 month, >1 month)	0.4876
Operative treatment	
Reduction (adequate/not adequate)	0.0443 *
Locking plate vs. Non-locking plate	0.3124
Time of surgery (<90 vs. 90-180 vs. >180)	0.0102 *
Mobilization	
Mobilization (<3 days vs. >1 week)	0.6811
Postoperative treatment	
Ambulatory physiotherapy (whether or not)	0.7072
CPM	0.0164 *
